# Constitutive Activation of Gli2 Impairs Bone Formation in Postnatal Growing Mice

**DOI:** 10.1371/journal.pone.0055134

**Published:** 2013-01-30

**Authors:** Kyu Sang Joeng, Fanxin Long

**Affiliations:** 1 Department of Medicine, Washington University School of Medicine, St. Louis, Missouri, United States of America; 2 Division of Biological and Biomedical Sciences, Washington University School of Medicine, St. Louis, Missouri, United States of America; 3 Department of Developmental Biology, Washington University School of Medicine, St. Louis, Missouri, United States of America; Hospital for Sick Children, Canada

## Abstract

Indian hedgehog (Ihh) signaling is indispensable for osteoblast differentiation during endochondral bone development in the mouse embryo. We have previously shown that the Gli2 transcription activator critically mediates Ihh function in osteoblastogenesis. To explore the possibility that activation of Hedgehog (Hh) signaling may enhance bone formation, we generated mice that expressed a constitutively active form of Gli2 in the Osx-lineage cells. Unexpectedly, these mice exhibited severe osteopenia due to a marked decrease in osteoblast number and function, although bone resorption was not affected. Quantitative analyses of the molecular markers indicated that osteoblast differentiation was impaired in the mutant mouse. However, the osteoblast-lineage cells isolated from these mice exhibited more robust osteoblast differentiation than normal *in vitro*. Similarly, pharmacological stimulation of Hh signaling enhanced osteoblast differentiation from Osx-expressing cells isolated from the wild-type mouse. Thus, even though Hh signaling directly promotes osteoblast differentiation in vitro, constitutive activation of this pathway impairs bone formation *in vivo*, perhaps through an indirect mechanism.

## Introduction

The Hedgehog (Hh) family of secreted proteins critically regulates developmental processes from flies to humans [1]. The mammalian genome encodes three Hh proteins, namely Indian hedgehog (Ihh), Sonic hedgehog (Shh), and Desert hedgehog (Dhh). Ihh is essential for endochondral skeletal development during mouse embryogenesis, as Ihh^−/−^ embryos exhibit severe defects in chondrocyte development, and fail to form osteoblasts, resulting in neonatal lethality [2]. Similarly, Ihh critically controls endochondral skeletal development in the chicken embryo [3]. Mouse genetic experiments with *Smoothened*, which encodes a seven-pass transmembrane protein indispensable for transducing intracellular Hh signaling, have demonstrated that Ihh directly controls chondrocyte proliferation as well as osteoblast differentiation from the perichondrial progenitors [4], [5]. In addition, Ihh controls the orderly progression of chondrocyte maturation both through the regulation of PTHrP and by direct mechanisms [6], [7], [8], [9]. The multiple roles of Ihh are mediated by the Gli family of transcription factors. Whereas derepression of the Gli3 repressor is primarily responsible for Ihh function in chondrocyte proliferation and maturation, activation of the Gli2 transcription factor is critical for Ihh-induced osteoblast differentiation [10], [11], [12], [13]. In addition, Gli1 may also participate in osteogenic program in response to Ihh [14]. Thus, extensive studies in the mouse embryo have established that Ihh critically controls osteoblast differentiation during endochondral skeletal development.

Several studies have implicated Hh signaling in regulating the postnatal skeleton. Inducible deletion of *Ihh* in chondrocytes in newborn mice caused growth plate disruption and trabecular bone loss at a later stage [15]. Forced-activation of Hh signaling in mature osteoblasts via *Ptch1* deletion caused osteopenia due to increased bone resorption [16]. However, others have shown that upregulation of Hh signaling in the *Ptch1* ^+/−^ mice increased trabecular bone mass due to enhanced osteoblast differentiation [17]. Thus, the effect of Hh activation on postnatal bones remains to be fully elucidated.

Here, we activate Hh signaling in the osteoblast lineage by expressing a constitutively activated form of the Gli2 transcription factor (ΔNGli2). We report that constitutive Hh activation suppresses bone formation in postnatal mice.

## Materials and Methods

### Mouse Strains

*R26-ΔNGli2*, *Osx-Cre* and *R26-mT/mG* mice are as previously described [11], [18], [19]. Usage of mice was approved by Washington University Animal Studies Committee.

### Expression Studies

In situ hybridization for osteoblast markers in embryonic or newborn mice were performed as previously described [11]. To assess mRNA expression in osteoblast-lineage cells associated with the bone surface in postnatal mice, total RNA was extracted with Trizol from tibias of six-week-old mice that were cleanly dissected and cleared of the marrow. The RNA was then subjected to RT-qPCR. For GFP detection in E18.5 embryos, the limbs were dissected and fixed with 4% paraformaldehyde (PFA) for 30 minutes and rinsed with PBS. After decalcification for one day with 14% EDTA, the limbs were sectioned with a cryostat at 10 µm thickness. For GFP detection in postnatal mice, tibias or femurs were fixed with 4% PFA for 1.5 hours, and decalcified for three days with 14% EDTA before cryostat sectioning. GFP was visualized under a fluorescence microscope (Leica).

### *In vitro* Osteoblast Differentiation Assays

To isolate osteoblast-lineage cells from the long bones, tibias were dissected clean and cut into small pieces with scissors. The bone fragments were then digested with collagenase for one hour at 37°C, rinsed with saline before being cultured in DMEM containing 10% bovine serum. After one week, cells that had migrated from the bone fragments and populated the culture dish were harvested and reseeded onto 6-well plates at 3×10^5^ cells per well. In some experiments, the cells were sorted for GFP expression (from the Osx-Cre allele) by FACS before reseeding. The cells were further cultured in an osteogenic media (DMEM, 10% bovine serum, 50 mM β-glycerol-2-phosphate and 50 µg/ml ascorbic acid) for six days before harvested for RNA extraction. RNA was analyzed for expression of osteoblast markers by RT-qPCR.

For CFU-OB assays, bone marrow stromal cells were harvested and cultured at low density as previously described [20]. Briefly, single-cell suspensions were seeded on T25 flasks and incubated in osteogenic media (DMEM, 10% bovine serum, 50 mM β-glycerol-2-phosphate and 50 µg/ml ascorbic acid) for two weeks without change of medium. The cells were then stained for alkaline phosphatase activity according to an established protocol. Clusters with 20 or more cells were considered colonies. Colonies containing >80% AP^+^ cells that exhibit a spread-out morphology were counted as CFU-OB.

### Analyses of Postnatal Bones

X-ray radiography of bones was conducted with a Faxitron X-ray system, and µCT analyses with a Scano Medical µCT system. For dynamic histomorphometry, alizarin red and calcein were injected into mice on 7 and 2 days, respectively, before harvest. Plastic sections were prepared by Musculoskeletal Research Core (Washington University), and analyzed for bone formation parameters with Osteomeasure Analysis System (Osteometrics). Osteoblast and osteoclast numbers were obtained by using Osteomeasure from paraffin sections of decalcified bones stained with TRAP and counterstained with hematoxylin. Paraffin embedding was performed by the Developmental Biology Histology Core (Washington University). Total bone resorption activity (CTX-I) was measured with the RatLaps ELISA kit (Nordic Bioscience Dignostics).

### Statistical Analyses

All quantitative data was subjected to student t-test with a minimum of three independent samples. P≤0.05 was considered statistically significant.

## Results

### Activation of Hh Signaling in Osx-lineage Cells Results in Severe Osteopenia

**Figure 1:**
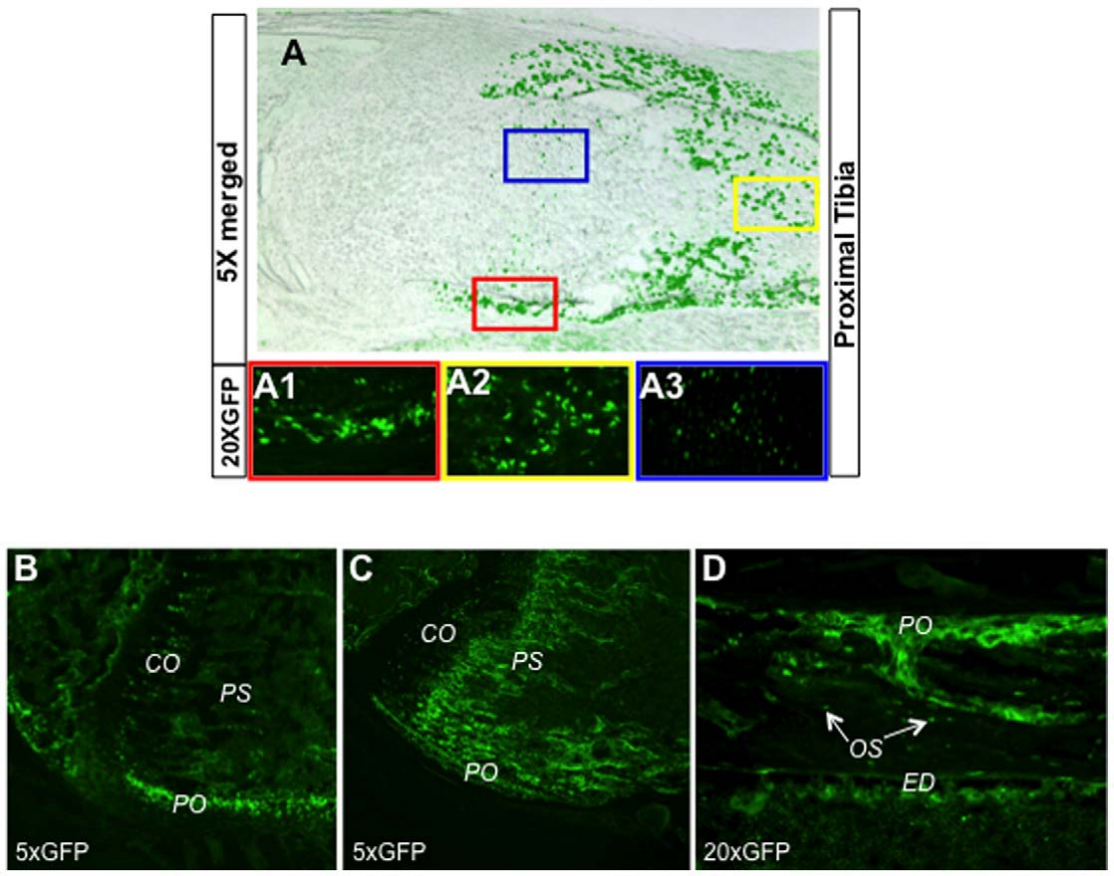
Characterization of *Osx-Cre* in embryonic and postnatal mice. (A) Detection of GFP in a section through the proximal end of tibia of *Osx-Cre* mice at E18.5. Shown is the merged picture from bright-field and green fluorescence images of the same section. (A1–A3) Higher-magnification green fluorescence images of areas in color-coded boxes in A representing perichondrium (A1), primary spongiosa (A2) and columnar chondrocytes (A3). (B, C) GFP detection in sections through the distal femur of *Osx-Cre* (B) or *Osx-Cre;R26-mT/mG* mice at 6 weeks of age (C). (D) GFP detection in a section through the tibial cortex of an *Osx-Cre; R26-mT/mG* mouse at 6 weeks of age. Images at a higher magnification in D than B and C. CO: columnar chondrocytes; PO: periosteum; PS: primary spongiosa; OS: osteocytes; ED: endosteum.

To activate Hh signaling in the osteoblast lineage, we set out to express the constitutively active form of Gli2 (ΔNGli2) from the *R26-ΔNGli2* allele that we have previously generated [11]. To this end, we first verified the utility of the *Osx-Cre* mouse line that expresses Cre fused with nuclear GFP from the regulatory sequences of *Osx*, which encodes a transcription factor essential for osteoblast differentiation [18]. As expected, GFP, which appeared to be within the nuclear compartment, was detected in osteoblast lineage cells within both the perichondrium and the primary spongiosa of the long bones at E18.5 (Figure 1A, A1, and A2). However, nuclear GFP was also observed in some columnar chondrocytes (Figure 1, A3), with a pattern similar to endogenous *Osx* expression in those cells [10]. In six-week-old mice, nuclear GFP was detected at the highest level in the perichondrium, and at a lower level in columnar chondrocytes as well as the primary spongiosa (Figure 1B). To detect the progenies of the cells targeted by *Osx-Cre*, we generated mice with the genotype of *Osx-Cre; R26-mT/mG*, wherein membrane-tethered GFP was expressed from the *R26-mT/mG* allele in cells expressing Cre, and their descendents. Here, in addition to nuclear GFP in the periosteum and the columnar chondrocytes, membrane-tethered GFP was abundant within the primary spongiosa, indicating that a majority of the cells associated with the primary spongiosa were originated from Osx-expressing cells (Fig. 1C). A closer examination of the cortical bone revealed that virtually all cells at the periosteum, endosteum, and the osteocytes were of the Osx-lineage (Fig. 1D). Thus, Osx-Cre effectively targets osteoblast-lineage cells both in the embryo and in postnatal mice.

**Figure 2:**
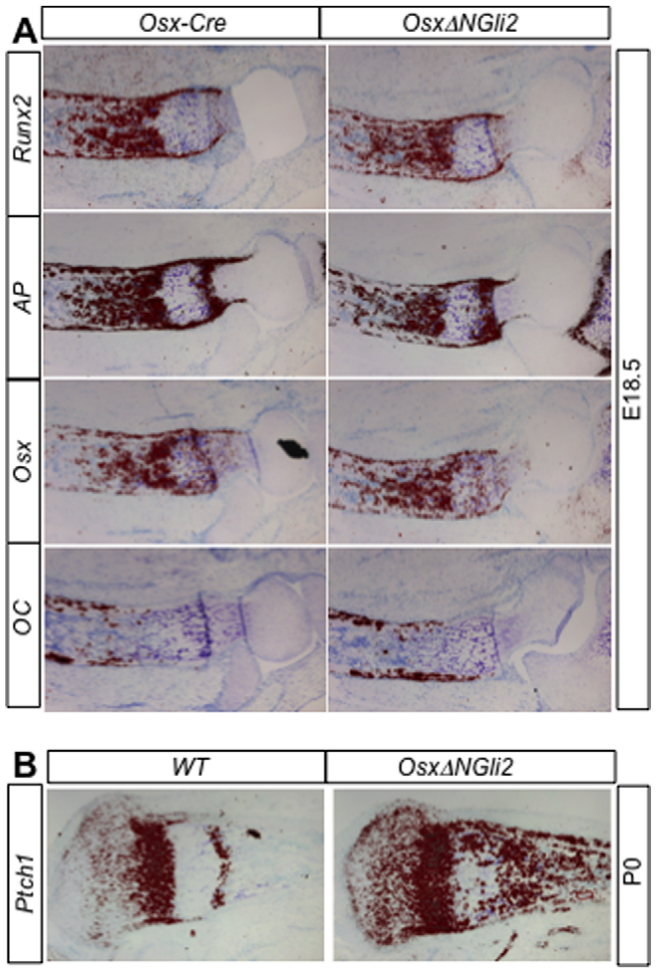
*In situ* hybridization on longitudinal tibial sections. Signal in red. Distal end to the right. (A) Expression of osteoblast markers in *Osx-Cre* versus *OsxΔNGli2* embryos at E18.5. (B) Expression of *Ptch1* in wild type (*WT*) versus *OsxΔNGli2* mice at P0.

Having confirmed that Osx-Cre targeted the osteoblast lineage, we generated *Osx-Cre; R26-ΔNGli2* mice (hereafter *OsxΔNGli2*) by crossing the *R26-ΔNGli2* and the *Osx-Cre* mice. We have previously shown that the *R26-ΔNGli2* mice exhibit no phenotype [11]. No obvious bone phenotype was observed with *OsxΔNGli2* mice during embryogenesis and at birth. In situ hybridization confirmed that all molecular markers for the osteoblast lineage, including *Runx2*, *alkaline phosphatase* (*AP*), *Osx* (also known as *Sp7*) and *osteocalcin* (*OC*) were expressed normally at E18.5 (Fig. 2A). On the other hand, expression of *Ptch1*, a direct transcriptional target of Gli proteins, was greatly expanded in the perichondrium/periosteum and the primary spongiosa of the *OsxΔNGli2* embryo, confirming the hyperactivity of Hh signaling in the osteoblast lineage (Fig. 2B). Thus, forced activation of Hh signaling in Osx-lineage cells does not grossly affect embryonic bone development.

**Figure 3:**
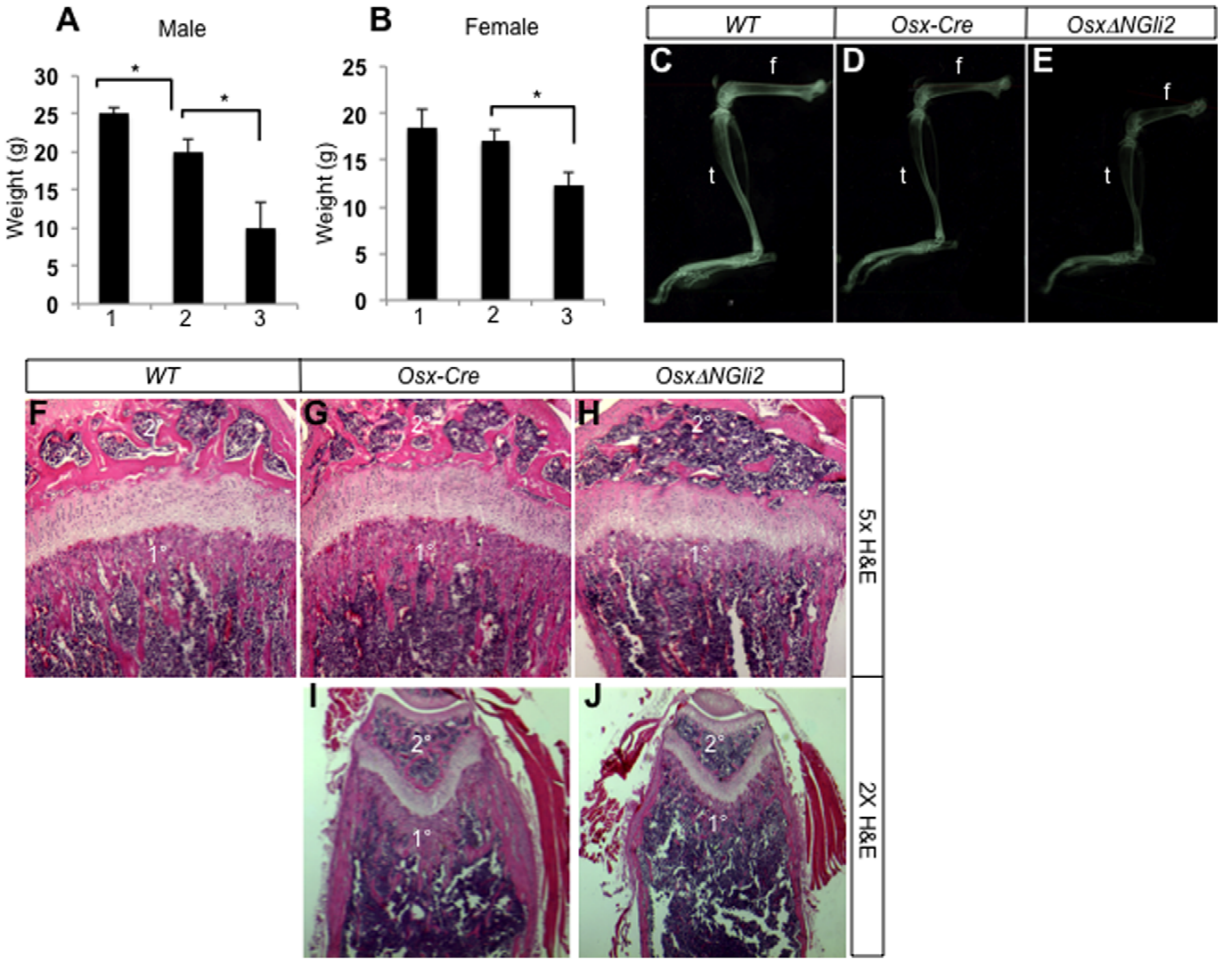
Growth defects and osteopenia in *OsxΔNGli2* mice at 6 weeks of age. (A–B) Body weights of males (A) or females (B) with indicated genotypes. 1: wild type; 2: *Osx-Cre*; 3: *OsxΔNGli2*. n = 3; *: p<0.05; error bar: STDEV. (C–E) X-ray radiography for the hindlimbs of wild type (C), *Osx-Cre* (D) and *OsxΔNGli2* (E) male mice at 6 weeks of age. f: femur; t: tibia. (F–H) H&E staining of sections from the proximal ends of tibias in 6-week-old male mice. (I, J) H&E staining of sections from the distal ends of femurs in 6-week-old male mice. 1°: primary ossification center; 2°: secondary ossification center. Note decrease in bone in both centers of *OsxΔNGli2* mice.

**Figure 4:**
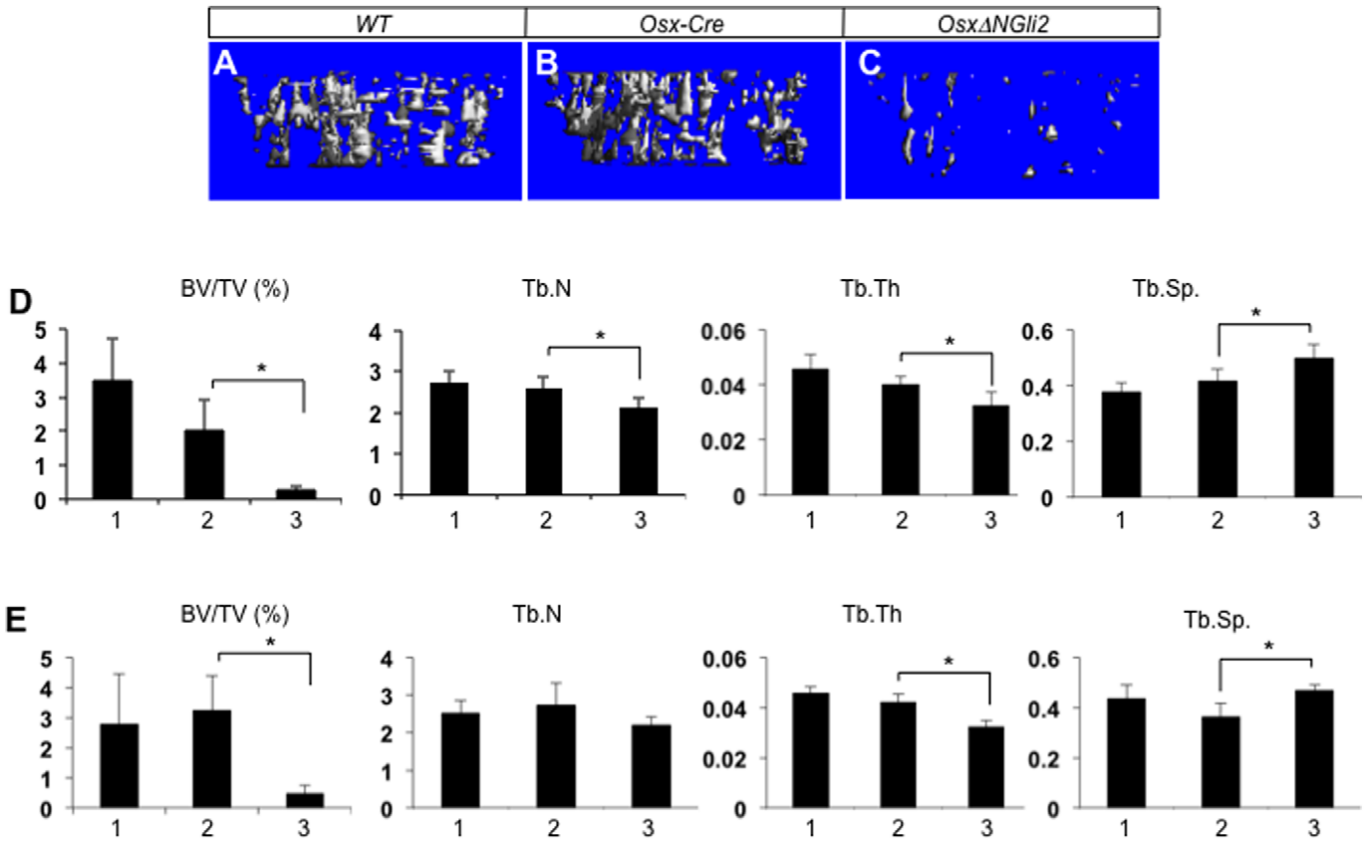
µCT analyses of trabecular bone in the proximal tibia of 6-week-old mice. (A–C) Examples of 3-D reconstruction images of the primary spongiosa in male mice with indicated genotypes. (D–E) Bone morphometric parameters from µCT analyses of the primary spongiosa in males (D) or females (E). 1: wild type; 2: *Osx-Cre*; 3: *OsxΔNGli2*. n = 3; *: p<0.05; error bar: STDEV.

We next examined the postnatal mice for potential bone phenotypes. The *OsxΔNGli2* mice of either sex showed retardation in postnatal growth, resulting in a notable reduction in body weight at 6 weeks of age when compared to their control littermates (Fig. 3A, B). Consistent with a previous report, the *Osx-Cre* males showed a slightly lower body weight than the wild type littermates, but we did not see a similar phenomenon with the *Osx-Cre* females [21] (Fig. 3A, B). X-ray radiography confirmed that the *OsxΔNGli2* mice of either sex possessed shorter long bones than either wild type or *Osx-Cre* sex-matched counterparts (Figure 3C-E, and data not shown). X-ray also revealed that the *OsxΔNGli2* mice of either sex possessed less bone than the sex-matched wild type or *Osx-Cre* controls, and that the *Osx-Cre* males but not females exhibited a variable degree of bone reduction when compared to the wild type control (Figure 3C–E, and data not shown). Histological analyses of the long bones confirmed a notably lower bone mass in the *OsxΔNGli2* mice than either wild type or *Osx-Cre* controls (Figure 3F–J). The low bone mass was evident at the primary and secondary ossification centers, as well as the cortex. On the other hand, the growth plate in *OsxΔNGli2* mice was relatively normal. Finally, µCT analyses showed that the trabecular bone mass (BV/TV) was markedly reduced in the *OsxΔNGli2* mice when compared to wild type or *Osx-Cre* mice, in both males and females (Fig. 4). The reduced bone mass was associated with reduced trabecular number and thickness, and increased trabecular spacing. Overall, expression of ΔNGli2 in the Osx-lineage causes severe osteopenia in the postnatal mice.

### Activation of Hh Signaling Decreases Osteoblast Number and Activity

**Figure 5:**
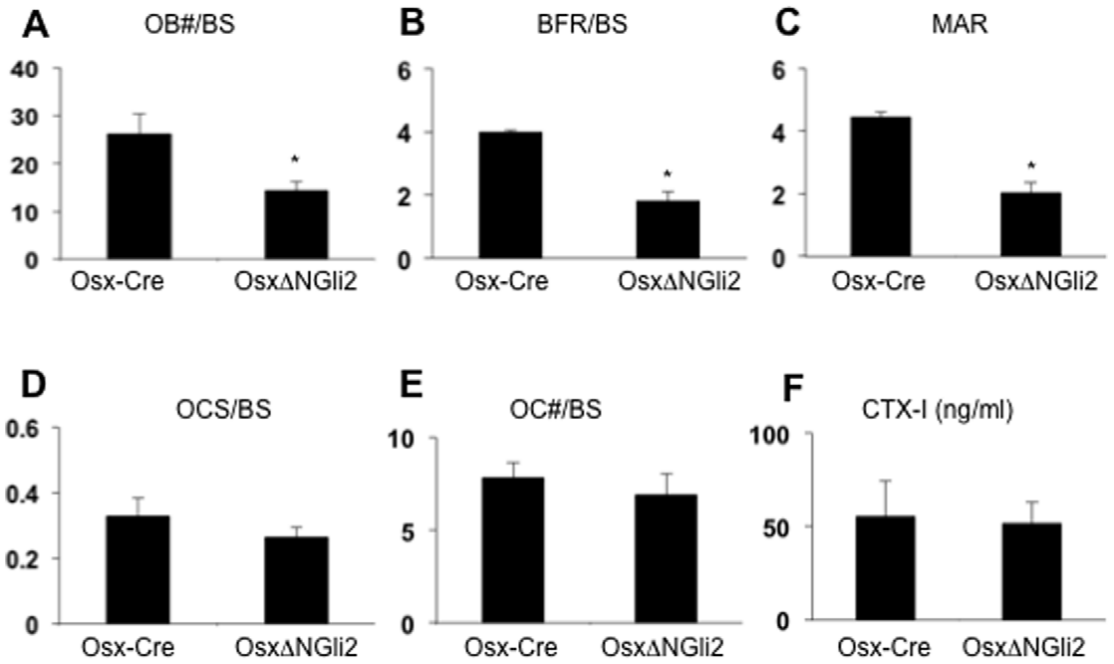
Analyses of osteoblasts and osteoclasts in 6-week-old male mice. (A) Osteoblast number normalized to bone surface. (B-C) Bone forming rate per bone surface (B) and mineral apposition rate (C) from dynamic histomorphometry. (D-E) Osteoclast surface (D) and number (E) normalized to bone surface. (F) Serum CTX-I assays.

To address the cellular basis for osteopenia in the *OsxΔNGli2* mice, we performed histomorphometric analyses. We observed a significant decrease in osteoblast number per bone surface, and a much reduced rate for mineral apposition and for bone formation in the *OsxΔNGli2* mice (Fig. 5A–C). On the other hand, there was no significant difference in osteoclast number or resorption surface between *OsxΔNGli2* and the control mice (Fig. 5D, E). We also detected no difference in the serum level of CTX-I, an indicator of total osteoclast activity, between the different genotypes (Fig. 5F). Therefore, osteopenia in the *OsxΔNGli2* animals is caused by reduced osteoblast number and activity, not changes in osteoclasts.

### Constitutive Hh Activation Confers Opposite Effects on Osteoblast Differentiation *in vivo* Versus *in vitro*

**Figure 6:**
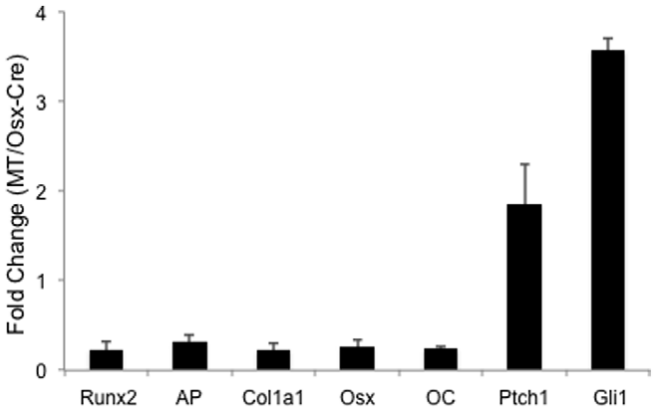
Relative expression of osteoblast markers and Hh target genes in bone-surface cells in 6-week-old *OsxΔNGli2* (MT) versus *Osx-Cre* male mice. Data by qPCR from either genotype was first normalized to *GAPDH* before fold changes were calculated. n = 3; error bar: STDEV.

The reduced osteoblast number in the *OsxΔNGli2* mice prompted us to examine whether osteoblast differentiation was impaired *in vivo*. For this, we extracted RNA directly from cells associated with the bone surface of the long bones and determined the expression levels of osteoblast differentiation markers by qPCR. These experiments revealed that *Runx2*, *AP*, *Col1a1*, *Osx* and *OC* were all markedly reduced in the *OsxΔNGli2* mice, whereas *Ptch1* and *Gli1*, both direct targets of the Gli transcription factors, were upregulated as expected (Fig. 6). Thus, impaired differentiation is at least in part responsible for the osteoblast deficiency in the *OsxΔNGli2* mice.

**Figure 7:**
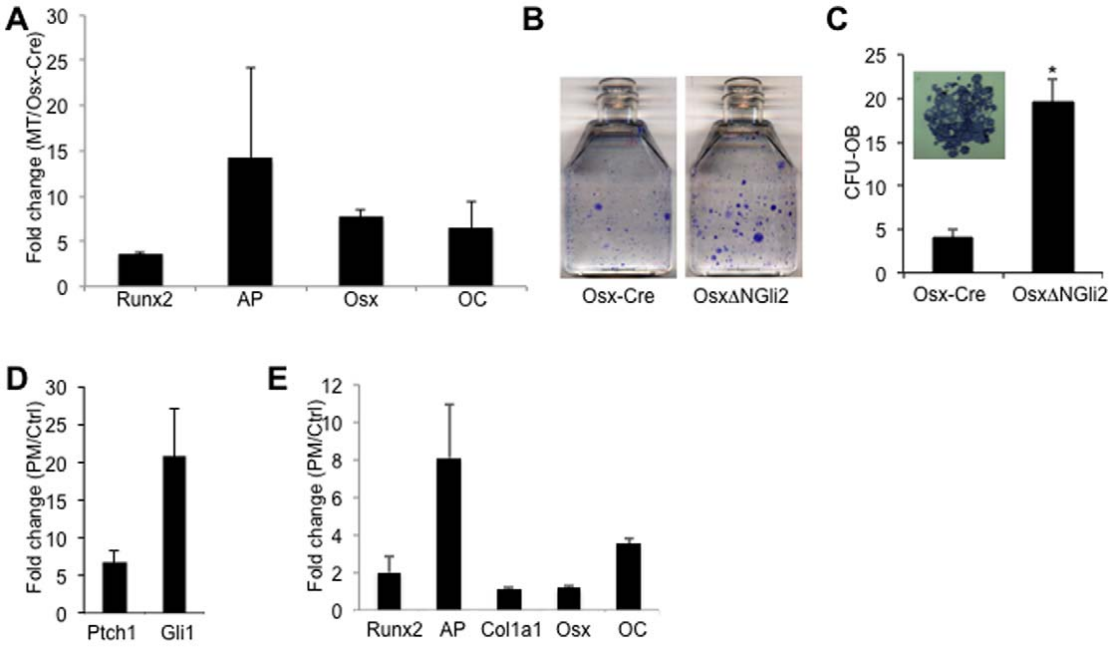
Osteoblast differentiation *in vitro*. (A) Relative expression of osteoblast markers in cultures of cells isolated from bone chips of 6-week-old *OsxΔNGli2* (MT) versus *Osx-Cre* male mice. Data by qPCR from either genotype was first normalized to *GAPDH* before fold changes were calculated. n = 3; p<0.05; error bar: STDEV. (B) Representative image of CFU-OB assays from 6-week-old male mice. Colonies visualized by AP activity staining. (C) Quantification of CFU-OB. A typical CFU-OB shown in inset. Shown is average from three flasks for each genotype. *: p<0.05. (D–E) Relative expression of Hh target genes (D) or osteoblast markers (E) in cultures of sorted Osx-expressing cells from bone chips of *Osx-Cre* mice, with treatment of purmorphamine (PM) or vehicle (Ctrl). Data by qPCR from either genotype was first normalized to *GAPDH* before fold changes were calculated. n = 3; p<0.05; error bar: STDEV.

To assess whether the impairment in osteoblast differentiation in the *OsxΔNGli2* mice was directly due to Hh activation in the differentiating cells, we isolated primary cells from the bones of *OsxΔNGli2* versus *Osx-Cre* mice, and examined their differentiation *in vitro*. In one set of experiments, we cultured osteoblast-lineage cells from small fragments of long bones dissected from either *OsxΔNGli2* or *Osx-Cre* mice, and compared the expression of osteoblast markers following the incubation in an osteogenic medium. We found that cells from the *OsxΔNGli2* mice expressed markedly higher levels of *Runx2*, *AP*, *Osx* and *OC* (Fig. 7A). As expected, these cells expressed much higher levels of *Ptch1* and *Gli1* when compared to the control (data not shown). In a second set of experiment, we cultured bone marrow stromal cells (BMSC) at a low density and evaluated the number of total versus osteoblast colonies (CFU, CFU-OB, respectively, see Materials and Methods for definitions) in the osteogenic media. Although CFU was similar between the genotypes, the number of CFU-OB was significantly higher in the cultures from the *OsxΔNGli2* mice (Fig. 7B, C). This result indicates that the BMSC cultures from *OsxΔNGli2* mice contained a normal number of progenitors, but these progenitors appeared to undergo accelerated differentiation *in vitro*. Overall, both sets of experiments support the conclusion that Hh activation directly stimulates osteoblast differentiation *in vitro*.

To address specifically the effect of Hh activation in Osx-expressing cells *in vitro*, we isolated Osx-expressing cells from the long bones by taking advantage of the fact that the *Osx-Cre* transgene expresses GFP. Briefly, cells isolated from small pieces of long bones dissected from *Osx-Cre* mice were sorted for GFP expression by FACS, and the GFP-positive cells were then cultured with or without purmorphamine (PM), a known stimulator of Hh signaling [22]. As expected, PM upregulated the expression of *Ptch1* and *Gli1* (Fig. 7D). Importantly, PM increased the mRNA levels of *Runx2*, *AP* and *OC*, without affecting those of *Col1a1* and *Osx* (Fig. 7E). Taken together, multiple lines of evidence indicate that activation of Hh signaling directly promotes osteoblast differentiation *in vitro*.

## Discussion

We have investigated the effect of sustained hedgehog signaling on bone formation. We report that expression of a constitutively active form of Gli2 (ΔNGli2) in the Osx-expressing cells and their progenies leads to osteopenia in postnatal growing mice, due to the suppression of osteoblast number and activity.

The inhibition of bone formation by Hh signaling is unexpected. Cultures of primary cells isolated from the ΔNGli2-expressing bones displayed more robust osteoblast differentiation than normal. Sorted Osx-expressing cells also underwent greater osteoblast differentiation in response to Hh stimulation. These *in vitro* findings are in agreement with previous studies showing that Hh signaling stimulates osteoblast differentiation in both cell lines and primary cell cultures [17], [23]. Nevertheless, we observed a notable decrease in the number of osteoblasts in the *OsxΔNGli2* mice, and molecular analyses indicated that osteoblast differentiation was impaired in those mice. The disconnection between the *in vivo* and *in vitro* findings may be explained by the fact that our strategy targeted not only pre-osteoblasts that actively express *Osx*, but also all of their progenies, including mature osteoblasts and osteocytes. It is possible that Hh activation in these other cell types indirectly suppresses osteoblast differentiation *in vivo* through either secreted factors or cell-cell contact. It is also possible that Hh stimulation in the osteoblast lineage activates an inhibitory feedback mechanism in another cell type *in vivo* that is absent *in vitro*. Future studies are necessary to distinguish these potential mechanisms.

Our study does not support an indirect regulation of osteoclastogenesis by Hh signaling in osteoblasts. A previous study reported that Hh activation in mature osteoblasts by deleting *Ptch1* with *OC-Cre* stimulated osteoclastogenesis in postnatal mice [16]. The reason for the discrepancy is not clear but could reflect the difference between the Cre lines. In addition, removal of *Ptch1* in the previous study might have effects mediated by effectors other than the Gli2 activator that was employed in the present study. For instance, it was shown that *Ptch1* haploinsufficiency reduced the level of Gli3 repressor in osteoblast lineage cells [17]. It is conceivable that different downstream effectors mediate distinct effects of Hh activation in the osteoblast lineage.

The physiological role of endogenous Hh signaling in postnatal bones warrants further study. Genetic deletion of *Smoothed* in mature osteoblasts was reported to increase bone mass in one-year-old but not two-month-old mice [16]. However, inhibition of Hh signaling by cyclopamine in eight-week-old male mice reduced bone mass due to decreased osteoblast number and function [17], whereas a similar treatment of eight-week-old female mice increased bone mass due to the suppression of bone resorption [24]. These seemingly contradicting results could indicate that the effect of Hh signaling within the postnatal bone microenvironment is exquisitely dependent on the level or duration of the signal, the target cell types as well as the systemic factors. Future studies that manipulate Hh signaling at specific stages of osteoblast or osteoclast lineage may help to unravel the complex roles in postnatal bones.
